# Supplementary material for: The causal role of circulating inflammatory markers in osteoporosis: a bidirectional Mendelian randomized study
Source: Front Immunol. 2024 Jul 18;15:1412298. doi: 10.3389/fimmu.2024.1412298 (PMC11291241; doi:10.3389/fimmu.2024.1412298)
Supplement: Supplementary file 1 [file DataSheet_1.zip › supplementary material/OSTEOPOROSIS-CIM/GCST90274808.tsv.gz_leave_one_out.pdf]

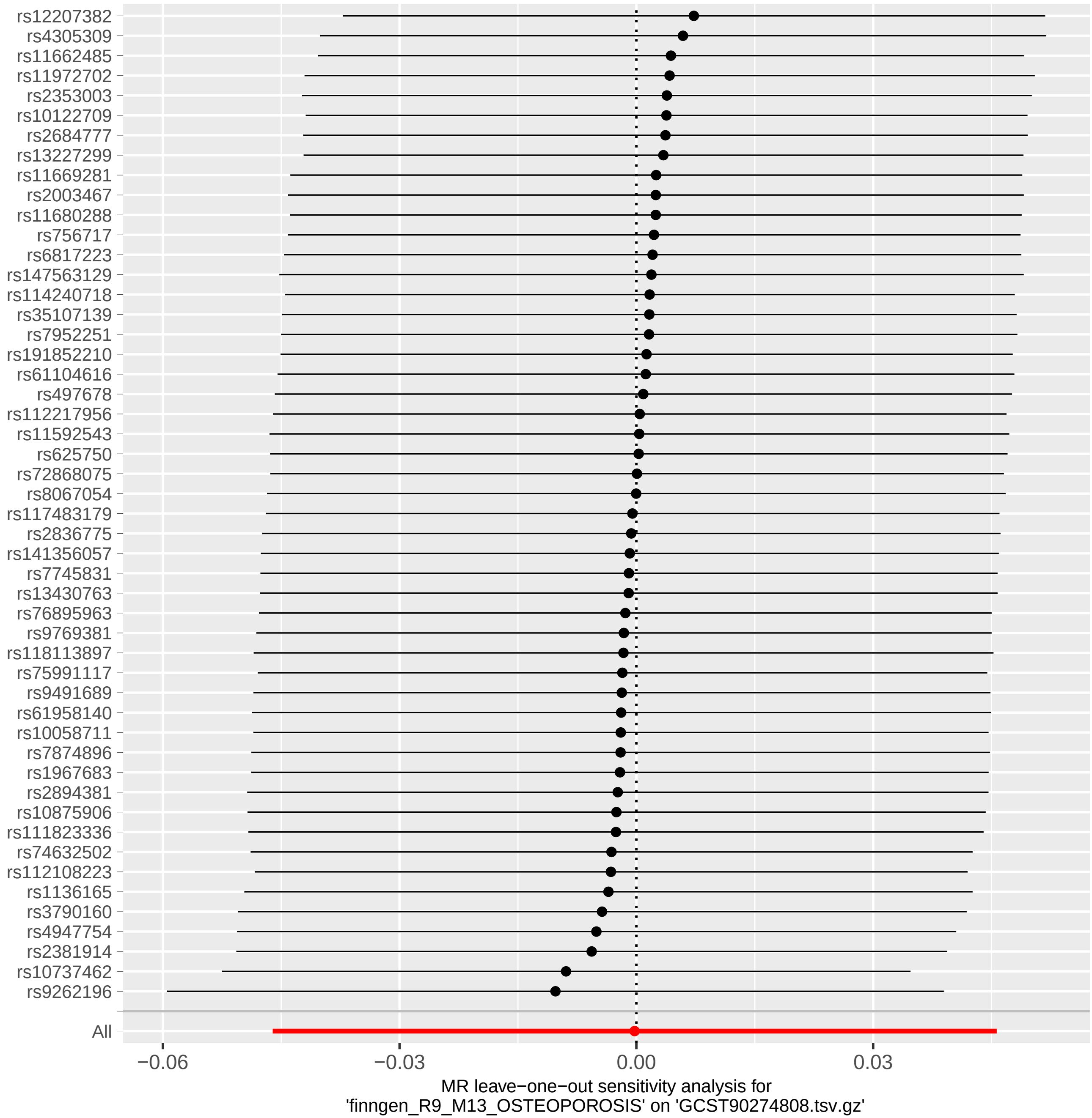
rs12207382
rs4305309
rs11662485
rs11972702
rs2353003
rs10122709
rs2684777
rs13227299
rs11669281
rs2003467
rs11680288
rs756717
rs6817223
rs147563129
rs114240718
rs35107139
rs7952251
rs191852210
rs61104616
rs497678
rs112217956
rs11592543
rs625750
rs72868075
rs8067054
rs117483179
rs2836775
rs141356057
rs7745831
rs13430763
rs76895963
rs9769381
rs118113897
rs75991117
rs9491689
rs61958140
rs10058711
rs7874896
rs1967683
rs2894381
rs10875906
rs111823336
rs74632502
rs112108223
rs1136165
rs3790160
rs4947754
rs2381914
rs10737462
rs9262196
All
−0.06
−0.03
0.00
0.03
MR leave−one−out sensitivity analysis for
'finngen_R9_M13_OSTEOPOROSIS' on 'GCST90274808.tsv.gz'
